# Physical Noninvasive Attacks on Photoplethysmogram by Computer Controlled Blood Pressure Cuff

**DOI:** 10.3390/s23249764

**Published:** 2023-12-11

**Authors:** Kazuki Yoshida, Ryota Sawano, Masahiro Okamoto, Kazuya Murao, Shuhei Tsuchida, Tsutomu Terada

**Affiliations:** 1 Graduate School of Information Science and Engineering, Ritsumeikan University, 1-1-1 Nojihigashi, Shiga, Kusatsu 525-8577, Japan; kazuki.yoshida@iis.ise.ritsumei.ac.jp (K.Y.);; 2 Center for Interdisciplinary AI and Data Science, Ochanomizu University, 2-1-1 Otsuka, Bunkyo-ku, Tokyo 112-8610, Japan; tsuchida.shuhei@ocha.ac.jp; 3 Graduate School of Engineering, Kobe University, 1-1 Rokkodai-cho, Nada-ku, Hyogo, Kobe 657-8501, Japan; tsutomu@eedept.kobe-u.ac.jp

**Keywords:** wearable, pulse wave, attack, peak disappearance, upper arm pressure, smartwatch, heart rate, manipulation, control

## Abstract

Sensor data has been used in social security and welfare infrastructures such as insurance and medical care to provide personalized products and services; there is a risk that attackers can alter sensor data to obtain unfair benefits. We consider that one of the attack methods to modify sensor data is to attack the wearer’s body to modify biometric information. In this study, we propose a noninvasive attack method to modify the sensor value of a photoplethysmogram. The proposed method can disappear pulse wave peaks by pressurizing the upper arm with air pressure to control blood volume. Seven subjects experiencing a rest environment and five subjects experiencing an after-exercise environment wore five different models of smartwatches, and three pressure patterns were performed. It was confirmed in both situations that the displayed heart rate decreased from the true heart rate.

## 1. Introduction

Given that Internet of Things (IoTs) devices are connected to the Internet, they are in danger of direct attacks. Moreover, there are cases in which IoTs devices are used to attack servers as stepping stones. On the other hand, there is a threat of system function failure/stop and information leakage when malicious circuits called hardware trojans are contained in the design and manufacturing stages of integrated circuits (ICs). It is difficult to detect hardware trojans contained in ICs because they are smaller in size than the original ICs. Software and hardware threats to IoTs devices are urgent issues that need to be solved. Many companies, universities, and research institutes around the world are working on this issue.

Another threat to the sensors installed in IoT devices is the attack that causes the incorrect processing of sensor data by upstream devices that use the sensor data. For example, there is an attacking method [1] that causes image recognition to be incorrect and an attacking method [2] that causes sensor elements to output arbitrary waveforms by applying ultrasonic waves to them. Vitality [3] provided by Sumitomo Life Insurance Company is a health insurance program with a system for evaluating the points received from exercise, health checkups, and other efforts. This program allows subscribers to receive discounts on insurance premiums by uploading the results of health checkups and by converting exercise information collected from smartphones and wearable devices into points. The system automatically converts the data into points, so there is a risk of attackers attacking the sensors and trying to modify the data to obtain services unfairly. One of the threats to the sensor is to attack the wearer’s body and modify the biometric information. The sensor values are numerical values of the actual state of a body. The higher the sensor’s accuracy, the more correctly the information is converted into a value. Therefore, it is difficult to detect attacks based on sensor values only, even if the hardware, network, and cloud storage after the measurement are equipped with strong security.

In this paper, to clarify the possibility of attacks on wearable devices by modifying biometric information, we propose a physical noninvasive attack method on photoplethysmogram (PPG) using a computer-controlled blood pressure cuff. The proposed method can induce pulse wave peak disappearance by controlling the blood volume with air pressure by tightening the upper arm. In addition, we evaluate whether the proposed method can cause wrong values to be displayed by a heart rate measurement application on a commercially available smartwatch.The primary contributions of this study are as follows:

- We clarified the danger of attacks by modifying the biometric information on the device wearer and made people aware that pulse wave data can be easily manipulated.
- We implemented the proposed method in a device and showed that the proposed method can be used to attack PPG by displaying incorrect heart rate values in a smartwatch application.
- The proposed method will be used to generate specific pulse waveforms, which will involve basic research for use in interaction, communication, and other technologies.

The remainder of this paper is organized as follows. Section 2 introduces related studies. Section 3 describes the proposed method in detail. Section 4 describes evaluation experiments, and Section 6 summarizes this study.

## 2. Related Works

This section introduces studies on attack methods on sensors and IoT, as well as studies on using pulse waves.

### 2.1. Studies on Attack Methods on Sensors and IoT

Researching an attack on the object to be measured, Sharif et al. [4] proposed a technique to generate an accessory in the form of eyeglass frames that can effectively fool state-of-the-art facial recognition systems. By wearing the generated accessory, they showed that it can bypass the face recognition systems that are widely used for surveillance and access control, as well as impersonate other people. Yamada et al. [5] designed luminescent glasses that can avoid face detection by adding noise to the captured image, thereby making use of the fact that the image sensor of a camera is sensitive to near-infrared light. Eykholt et al. [1] proposed an algorithm that causes misclassification of image recognition results by attaching stickers to road signs, which are real-world measurement objects. By attacking real road signs using only black and white stickers, they showed that images captured in a laboratory environment and video frames captured in a moving vehicle caused 100% and 84.8% of misclassifications, respectively. Trippel et al. [2] proposed a method to generate arbitrary waveforms using ultrasound waves on accelerometer elements. The attack was performed on 20 models of capacitive accelerometers from five different manufacturers. A total of 75% of the output biases were shown to be weak. This means that an unsecured low-pass filter allows false variable output measurements under acoustic interference. In addition, they showed that 65% of the output controls are vulnerable. This means that an unsecured amplifier allows false output measurements under acoustic interference. Fujii et al. [6] proposed a method to misrecognize PPG sensor measurements by blinking a grayscale display screen to simulate the increase or decrease in blood volume. The results showed that the error of the displayed heart rate was ±3 bpm.

Farrukh et al. [7] proposed a side-channel attack method to identify what was written on an iPad with an Apple Pencil using the motion sensor data embedded in the iPad. The evaluation results on 10 subjects showed that the method correctly identified letters, numbers, shapes, and words in 93.9%, 96%, 97.9%, and 93.33% of cases, respectively. Gazzari et al. [8] propose a method for hacking EMG and IMU data by attacking the Myo armband to estimate keystrokes entered on a physical keyboard. The neural network was trained with labeled data for each character, and the trained model was used for estimation. The evaluation experiments of the proposed method showed that the mean balanced accuracy of keystroke detection was about 76%. Shen et al. [9] proposed VLA, a novel attack method for black-box face recognition systems using visible light, which was based on the difference in the image formation principles between a camera and the human eye. This approach creates adversarial perturbations based on visible light and projects them onto the human face, thereby allowing for targeted and nontargeted attacks.

Some attack methods have been proposed for objects, sensors, and people. However, to the best of the authors’ knowledge, there are no studies about how to attack the PPG by manipulating biometric data.

### 2.2. Studies on Using Pulse Waves

Many studies have used pulse wave data in various ways. Robert et al. [10] estimated the ECG peak time by calculating the peak-to-peak time difference from the pulse wave sensor data. They estimated the ECG peak with an average error of 14.3 ms in the standing position and 9.43 ms in the sitting position. Rajala et al. [11] evaluated the ability to calculate pulse wave arrival times from pulse waves measured at the wrist and fingers. The use of pulse wave data measured at rest in 30 subjects showed that the first derivative peak method was suitable for calculating pulse wave arrival time at the wrist. The results also show that the pulse waveforms of the wrist and fingers differ in shape and amplitude.

A method has been proposed for estimating blood pressure values from measured pulse wave data. Yoon et al. [12] tested whether blood pressure values can be continuously estimated in a cuffless environment. The method was estimated using pulse wave analysis with a multiparameter model. With an average regression model, the resulting standard deviation of error was 8.7 ± 3.2 mm Hg for the estimation of systolic blood pressure and 4.4 ± 1.6 mm Hg for diastolic blood pressure. Liu et al. [13] estimated blood pressure values using pulse waves measured by pressure sensors and a linear regression model. The linear regression model was trained using 21 different features that were extracted from the measured pulse wave. In addition, the system used a pressure sensor, which made it possible to estimate the cuffless values. The evaluation of 65 subjects in the middle and high age group showed that systolic blood pressure was estimated with an error of 1.33 ± 0.37 mm Hg, and diastolic blood pressure was measured with an error of 1.14 ± 0.20 mm Hg. Sun et al. [14] proposed a method for estimating systolic blood pressure values from electrocardiogram and pulse wave data. They extracted 18 types of features from the obtained data and estimated the blood pressure values by creating a multiple linear regression model. The evaluation experiment was conducted by collecting electrocardiogram and pulse wave data from 19 subjects who exercised for 40 min. As a result, the standard deviation in the error of the blood pressure value estimated by including the pulse wave arrival time data was 13.52 mm Hg.

A method for estimating the sensor attachment position using the pulse wave measured by the sensor was proposed. Yoshida et al. [15] proposed a method to estimate the sensor position based on the time difference between the ECG and pulse wave peaks, thereby taking advantage of the fact that the pulse wave arrival time differs for each body part. The results showed that the method was able to estimate the position of the sensor with 100% accuracy for 2–5 body parts. There are many methods for using measured pulse wave data. However, to the best of the our knowledge, no studies are focusing on pulse wave control.

The action of stopping blood flow is also performed during blood pressure measurement. Typical blood pressure measurement methods include the oscillometric method [16] and the Korotkoff method [17,18]. A cuff (armband) is wrapped around the upper arm, the air is pumped in to compress the blood vessels to stop the flow of blood, then the pressure of the blood exceeds the pressure of the cuff compressing the blood vessels when the pressure is gradually relaxed, and blood begins to flow intermittently in time with the heartbeat. The oscillometric method determines blood pressure values by measuring cuff pressure fluctuations (pressure pulse) that reflect the oscillations of the arterial wall in synchronization with the beating of the heart during the stages of cuff depressurization after cuff pressurization. Generally, the cuff pressure at which the pressure pulse increases suddenly is considered to be the maximum blood pressure, and the cuff pressure at which it decreases suddenly is considered to be the minimum blood pressure. On the other hand, the Korotkoff method uses a microphone in the cuff or a stethoscope to detect vascular sounds, which are called Korotkoff sounds (K-sounds) and are generated when blood begins to flow intermittently in relation to the heartbeat during the decompression phase after the cuff is pressurized. The maximum blood pressure is defined as the cuff pressure at the K-sound starting time, and the minimum blood pressure is defined as the cuff pressure at the K-sound disappearing time. Currently, electronic blood pressure monitors are mostly based on the oscillometric method. Thus, the pulse wave is controlled to be stopped during blood pressure measurement, but it is not controlled dynamically and intermittently, as is performed in this study.

## 3. Proposed Method

This section describes a method for controlling pulse waves with upper arm compression.

### 3.1. Effect of Upper Arm Compression on Pulse Wave Measurement

In this study, we assume that a user wears a wearable device such as a smartwatch equipped with a photoelectric pulse wave sensor (PPG sensor) on the user’s wrist. The photoelectric method uses an LED to irradiate light with a green wavelength near 550 nm onto the skin surface, and a photodiode on the skin surface receives the irradiated light reflected by body tissues. Since hemoglobin absorbs incident light, the pulse rate is measured by utilizing the fact that the reflected light decreases due to the increase in blood volume at the moment when the pulse wave arrives. Therefore, by compressing the upper arm and decreasing the blood volume, the sensor value becomes smaller at the end of the fingertips from the upper arm to the fingertips. Our research group has reported that the pulse wave is attenuated by sufficient pressure on the blood vessels [19].

**Figure 1:**
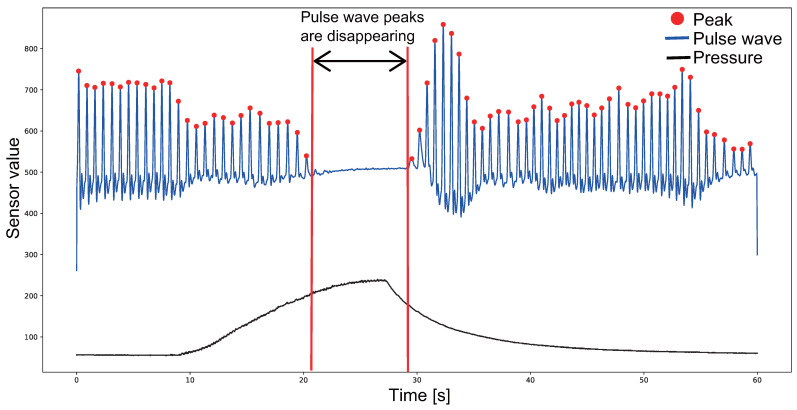
Pulse waveform of fingertip during compression of the upper arm. The vertical axis indicates the A/D-converted pulse wave and pressure sensor values, the horizontal axis indicates time, and the red circle indicates the pulse wave peaks.

Figure 1 shows the pulse waves of the pulse sensor values measured at the fingertips when the upper arm was pressed. Measurements were taken at the fingertips of the right hand, and the upper arm was pressed by attaching a cuff to the right upper arm and pumping air. A pulse sensor (made by https://pulsesensor.com/, accessed on 6 December 2023) [20] was attached to the fingertip of the right hand, and the pulse sensor value at the fingertip was observed for 60 s. Air was pumped into the cuff with the pressure sensor value increasing by 10 until the pressure sensor value reached 240, and air was exhausted with the pressure sensor value decreasing the value by 20. Figure 1 shows that the pulse wave sensor value became smaller as the pressure pressing on the upper arm increased, and the pulse wave peak disappeared after a constant pressure value was reached. In addition, when the air in the cuff is exhausted, the pulse wave peak can be detected again. Based on this fact, we designed a method that performs compression and release on the upper arm at an arbitrary time.

### 3.2. Device Configuration

**Figure 2:**
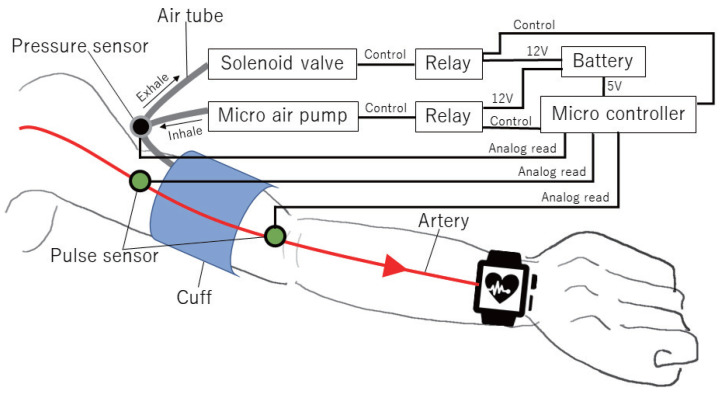
Configuration of devices to be implemented by the proposed method.

Figure 2 shows an overview of the proposed device. The device consists of a one-board micro controller, a circuit using mechanical relays, a compression cuff (hereinafter referred to as “cuff”), a micro air pump, a solenoid valve, pulse sensors, a pressure sensor, and a battery. The micro air pump and solenoid valve are connected to the cuff using tubing. The micro air pump blows air into the cuff to inflate it and compress the arm. The solenoid valve is used to vent the inside of the tube. The micro air pump and solenoid valve are controlled by a micro controller using a relay circuit. The pulse sensors are used to measure pulse waves at two points before and after passing through the proposed device. The pressure sensor is connected to the tube connected to the cuff and is used to measure the pressure value inside the cuff.

### 3.3. Overview of the Proposed Method

**Figure 3:**
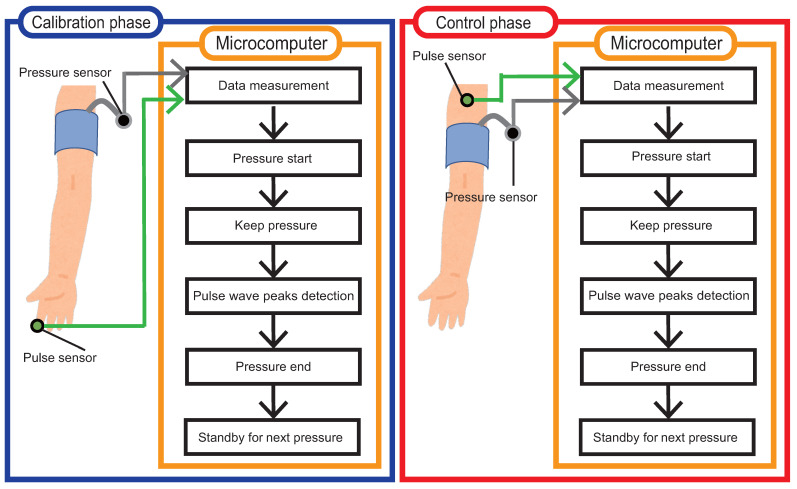
The process flow of the proposed method.

Figure 3 shows the process flow of the proposed method. As shown in Figure 3, the proposed method consists of two processing phases: calibration and control. Calibration is the phase that determines the suitable pressure value for the user and uses a pulse wave sensor attached closer to the fingertip than the cuffed position, as well as a barometric pressure sensor attached to the tube. After the calibration phase is finished, pulse wave control is performed. Control is the phase that actually controls blood flow, and it uses a pulse wave sensor attached closer to the heart than the cuffed position, as well as a pressure sensor attached to the tube. The calibration and control phases consist of six processes: data measurement, pressure start, keep pressure, pulse wave peaks detection, pressure end, and standby for next pressure. Each of these processes is described in detail in the following sections.

### 3.4. Calibration

We assume that the user wears the pulse sensor on the fingertip side of the cuff and that the pressure sensor is connected to the tube that is connected to the cuff. Calibration is performed to achieve the appropriate upper arm pressure for the user. The pressure value at which blood flow stops differs depending on the user’s body shape and age. Therefore, the calibration phase searches for the maximum pressure value at which the pulse wave peak has disappeared by pressing on the upper arm before the actual pulse wave control, and it calculates the pressure value suitable for the user.

Table 1 shows the execution procedures and contents of the calibration phase. The calibration phase consists of six steps. First, the pulse wave and pressure are measured every *T* [ms]. Note that *T* is a time that can be set arbitrarily. Hereafter, the pulse wave measurement at time *T* is referred to as p(t), and the pressure sensor value at time *T* is referred to as $x_{air}\left(t\right)$. Next, the solenoid valve is closed, and air is supplied from the micro air pump to start pressure. Here, the target pressure value for the pressure is set to $x_{air\_max}$. At the start of the calibration phase, $x_{air\_max}=400$ is set. Next, when $x_{air}\left(t\right)\geq x_{air\_max}$ is satisfied, the micro air pump stops pumping air. Thereafter, the peaks that appear in the measured pulse wave are detected using the peak detection algorithm [21]. The peak detection algorithm is designed to reset the threshold when no new peaks are detected in 2 s. Using this function, the pulse wave disappearing state is determined when the peak is not detected for 2 s. Note that the reason for setting 2 s is to detect that the peak has disappeared without fail, because the human resting heart rate is 60–70 bpm or higher, and the heartbeat interval is 0.85–1 s or less. After that, the solenoid valve is opened for 1 s to release the air in the cuff. Finally, when the pulse wave disappearing state is detected, we update the value to $x_{air\_max}=x_{air\_max}-20$ and repeat this procedure from the beginning. On the other hand, when the pulse wave disappearing state is not detected, the calibration phase is finished by updating the value to $x_{air\_max}=x_{air\_max}+20$.

### 3.5. Pulse Wave Control

Pulse wave control phase is performed using the normally measured pulse wave. Therefore, it is assumed that the user wears the pulse wave sensor on the heart side rather than the cuff and that the air pressure sensor is connected to the tube connected to the cuff. The process described in this section is repeated to enable pulse wave control over a long time.

Table 2 shows the execution procedures and contents of the pulse wave control phase. The pulse wave control phase consists of six steps. First, the pulse wave and pressure are measured every *T* [ms]. Note that *T* is a time that can be set arbitrarily. Next, the solenoid valve is closed, and air is pumped from the micro air pump until the pressure value satisfies $x_{air\_max}$. Next, when $x_{air}\left(t\right)\geq x_{air\_max}$ is satisfied, the micro air pump stops pumping air. At this time, the solenoid valve is closed, so the pressure value is maintained at $x_{air\_max}$, and the pulse wave disappearance state is continued. Therefore, assuming $N_{stop}$ as the number of pulse wave peaks disappears, the air pressure value is maintained until $N_{stop}$ peaks are detected. Thereafter, the peaks that appear in the measured pulse wave are detected using the peak detection algorithm [21]. After that, the solenoid valve is opened when $N_{stop}$ peaks are detected, and the air pressure sensor value is reduced until it reaches $x_{air}\left(t\right)=x_{air\_max}\times 0.5$. Finally, no pressure is kept until $N_{appear}$ peaks are detected. Note that $N_{appear}$ denotes the number of pulse wave peaks to appear. By repeating this process sequence, it is possible to control the pulse wave over a long time.

### 3.6. Implementation

A device was implemented to control pulse waves using upper arm compression. The cuff and air tube were supplied by the HCR-7202 made by Omron Corporation, Kyoto, Japan. A microcomputer was divided into a data measurement microcomputer and a relay control microcomputer (Arduino UNO) to avoid noise contamination in the sensor value due to relay switching. Each microcomputer was supplied with 5 V via a connection to a PC. The microcomputer for data measurement was connected to two pulse wave sensors, a pressure sensor, and a tact switch. The pulse wave sensor made by pulsesensor.com [20] was used, and the pressure sensor used an MIS-2500-015G [22] made by Metrodyne Microsystem Corp., Hsin-Chu, Taiwan. The tact switch used a TS-0606-F-N-BLK [23] made by Cosland Co., Taipei, Taiwan. The reason for setting up the tact switch is to enable the device to start and end operations at an arbitrary time. In addition, it is connected to a microcomputer for relay control to send the timing of relay switching. The microcomputer for relay control was connected to a micro air pump and a solenoid valve. The micro air pump was a 12 V air pump made by Garosa, and the solenoid valve was a 2V025 model made by Walfront. The micro air pump and solenoid valve were supplied 12 V power from a power unit (Sky Toppower STP3010D, Hong Kong, China). The relay was a Y14H-1C-5DS made by Hsinda Precision CO., LTD, Taipei, Taiwan.

## 4. Evaluation Experiment

This section describes an evaluation experiment when the pulse wave is controlled by pressure on the upper arm using the proposed method.

### 4.1. Experiment Environment

#### 4.1.1. Data Collection

**Figure 4:**
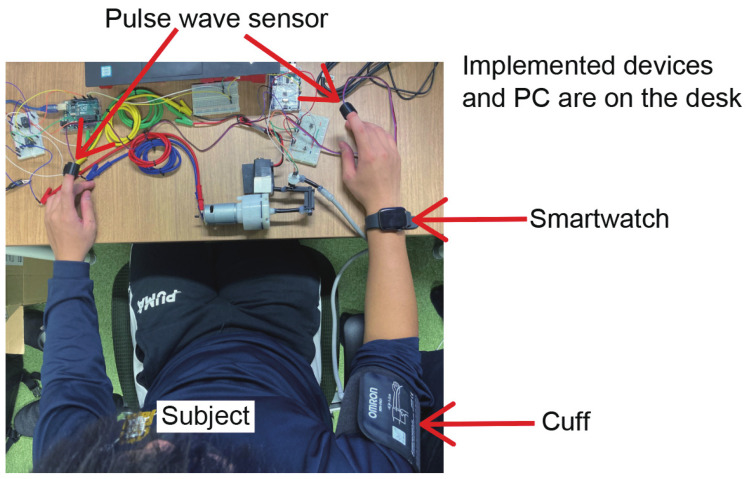
Scene of the subject during the experiment. Pulse sensors were attached to the index fingers of both hands. The pulse wave sensors were covered with black cloth tape to prevent the influence of ambient light. A cuff was attached to the right upper arm, and a smartwatch was attached to the right wrist (In this figure, an Apple device is worn). The implemented device and the PC are placed on the desk.

Seven subjects (A–G, all male, average age 22.8 years) pressed their upper arms with the implemented device. A cuff was attached to the subject’s right upper arm, and a smartwatch was worn on the right wrist. The smartwatch was worn in two situations: at rest and after exercise (after running for 1–2 min). The experiment was conducted with seven subjects at rest (A–G) and five subjects after exercise (A–E). The PPG sensors were attached to the index fingers of both hands to check whether the pressure correctly disappeared the pulse wave peaks. The PPG sensor values at the fingertips of both hands were recorded at 200 Hz. Figure 4 shows the scene of the subject during the experiment.

**Figure 5:**
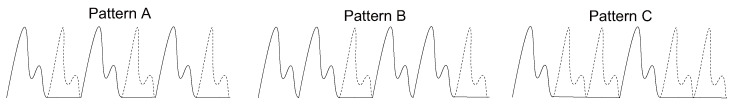
Pulse waveforms in three pressure patterns. The solid line indicates a appearing pulse wave peak, and the dotted line indicates a disappearing pulse wave peak.

The five smartwatches were selected: a Fitbit Versa 2 (Fitbit) [24], an Apple Watch Series 6 (Apple) [25], a Polar Ignite (Polar) [26], a Garmin Swim 2 (Garmin) [27], and a Paenoon full-touch screen smartwatch (Paenoon). Among the five models, the Fitbit, Apple, Polar, and Garmin were allowed to measure heart rate data and submit the measured data from the health insurance service [3]. The heartbeat monitor on each smartwatch was video-recorded for 2 min. The reason for using video recordings was that heart rate data could not be extracted from the smartwatch. The video-recorded data were manually transcribed, with the values displayed frame-by-frame at 1 s intervals. The calibration of the implementation device was performed in advance, and the 2 min period began when the pulse wave control was started. Figure 5 shows pulse waveform images of the upper arm pressure patterns of the device in this experiment. The solid line indicates appearing pulse wave peaks, and the dotted line indicates disappearing pulse wave peaks. Pattern A was set when the number of pulse wave peaks to be disappeared and to appear were the same, pattern B was set when the number of pulse wave peaks to appear was larger than the number of pulse wave peaks to be disappeared, and pattern C was set when the number of pulse wave peaks to appear was smaller than the number of pulse wave peaks to be disappeared. Pattern A was designed to investigate the limitations of the proposed device, and Patterns B and C were designed to investigate the versatility of the proposed method.

Each subject was measured twice per pressure pattern. As a result, a total of 720 min of data were collected from seven subjects × 2 min per experiment × three types of pressure patterns × five smartwatch models × two measurements = 420 min at rest, and five subjects × 2 min per experiment × three types of pressure patterns × five smartwatch models × two measurements = 300 min at after exercise.

#### 4.1.2. Evaluation Index

In this study, the evaluation index determines whether the heart rate displayed on the smartwatch screen can be reduced compared to the actual heart rate (BPM). To compare the displayed heartbeat on the smartwatch with the BPM, the BPM calculated from the value acquired by the pulse sensor attached to the left index fingertip was used as the true value in this experiment. The method of calculating the BPM from the pulse wave sensor values is described as follows. The pulse sensor values may include noise due to the condition of the user’s skin contact. To avoid detecting false pulse wave peaks, the moving average filter with a length of 41 samples (≈200 ms) was used, where the raw sensor data at time *t* were defined as p(t). The pulse wave value after applying the moving average filter at time *t* was determined as $p^{\prime }\left(t\right)=\sum_{i=t-20}^{t+20}p\left(i\right)$. Then, the peak detection was conducted as $p^{\prime }$. The peak detection was conducted using “find_peaks”, which is a Python Scipy (SciPy 1.7.3) [28] package. The “find_peaks” is a function that searches for the maximum value in the input data. The pulse wave peaks were detected using this function. In addition, the detected peaks can be controlled by setting parameters in “find_peaks”. In this experiment, the “height”, which sets the threshold for peak height, was set to the average of the pulse wave sensor values over 2 min. The “distance”, which sets the distance to not detect neighboring peaks in the time direction, was set to 100 at rest and 50 after exercise. The reason for changing the value for each environment was to detect the correct peak, even in environments with a high heart rate. After the peaks were detected, a visual check was conducted, and any inappropriate peaks were deleted.

After all the peaks were detected, the time difference between peaks was calculated. The *k*th peak from the youngest among the detected peaks is denoted by $p_{k}\left(k=1,\ldots \right)$, and the time of peak occurrence is denoted by $t_{k}$. The $d_{k}$ is denoted by the time difference between the peaks. The time difference between peaks can be calculated as $d_{k}=t_{k}-t_{k-1}$ from the $t_{k}$ and $t_{k-1}$ of the two *k*th and k−1th peaks in the detected peaks. The BPM (b(t) (0≤t≤T)) at *t* is calculated using the calculated time difference between the peaks according to the following Equation (1):

(1) $$b\left(t\right)=\left\{\begin{array}{cc} 60/\sum_{k=t=0}^{t}d_{k}/t & \left(t<10\right)\\ 60/\sum_{k=t-10}^{t}d_{k}/10 & \left(10\leq t\right) \end{array}\right.$$

The BPM is calculated by dividing 1 min (60 s) by the time difference between peaks $d_{k}$. When the BPM of a single peak is calculated, a big error occurs due to a failure in the peak detection. Therefore, the average of the time difference between 10 peaks was used. If k<10, the average value of the already-obtained data for *k* points was used, as dividing by 10 gives an incorrect average value (upper part in Equation (1)). The b(t) calculated by the above equation is the true value in the experiment.

### 4.2. Investigation of Limitations of Smartwatches in Displayed Heartbeat

#### 4.2.1. Experiment Environment

In this experiment, the upper and lower limits of the heart rate displayed on five smartwatches used in this study were investigated. For this purpose, the upper limit was investigated to see to what limit the heart rate can be displayed when the heart rate is high, such as after exercise. On the other hand, the lower limit was investigated to see to what limit the heart rate can be displayed when the pulse wave is controlled by pressure. To display the specified heartbeat rate, we used the Fujii et al. method [6]. Fujii’s method can display the specified heartbeat rate by flashing a grayscale display screen to simulate an up or down blood volume. We used the 3.5-inch displays developed for Raspberry Pi by Elecrow. The display was connected to a PC via an HDMI cable. A smartwatch was placed on the display, and the heartbeat rate was displayed using the heartbeat application installed in the smartwatch.

#### 4.2.2. Results

Table 3 shows the maximum and minimum heartbeats displayed for each smartwatch model. According to Machado et al. [29], they found that the maximum human heart rate can be calculated as 208 − (0.7 × age). Assuming a 20-year-old human wearing a smartwatch, Fitbit and Apple showed results with values above the maximum heart rate. The Garmin and Paenoon also displayed a value exceeding 180 BPM, which is considered to be sufficient for the heartbeat after exercise. Focusing on the minimum heartbeat, Apple, Garmin, and Paenoon displayed a value of approximately 45 BPM, and the Fitbit also displayed 54 BPM. In current medical practice, the minimum heart rate for adults at normal rest is 60 bpm, and the literature [30] that investigated this fact exists. The displayed value is below the normal resting heart rate. These results mean that smartwatches, except Polar, can handle the range of human heartbeats that can be measured normally.

In some cases, the heart rate of the PPG may contain an error of a few BPM. Compared to the ECG, which measures the electrical signals caused by the beating of the heart, the PPG measures changes in blood flow, so errors are caused by noise from arm movements or incorrect fitting of the device, such as a loose band. There is a study [31] comparing the accuracy of heart rate display values among the models sold by Apple, Fitbit, and Garmin, which are the experimental models in our study. According to this study [31], the Apple, Fitbit, and Garmin models displayed a heart rate with a ±3% error from the actual heart rate 71% of the time, 51% of the time, and 49% of the time, respectively. A ±3% error in the actual heart rate means that an 80 BPM heart rate is about 82 bpm or about 77 bpm, which shows that there is an error in the displayed value. This study could have caused errors larger than the ±3% error in the previous survey reference [31]. However, in this study, it was shown that the heart rate was reduced by 50% from the actual heart rate, i.e., the heart rate was displayed as 40 BPM for a person whose heart rate was 80 BPM. The details are described in Section 4.3 and Section 4.4; for example, it was shown that the heart rate was reduced by 50% from the actual heart rate, i.e., the heart rate was displayed as 40 BPM for a person whose heart rate was 80 BPM. On the other hand, Polar failed to display the heartbeat using the method of Fujii et al. This is because the Polar device cannot measure pulse waves unless the skin is in contact with the electrodes in the pulse wave sensor. Fujii et al.’s method does not require skin contact, and it is assumed that models that require skin contact can be supported by attaching a flexible display on the skin.

### 4.3. Results of Heart Rate Changes at Rest by Pulse Wave Control

Table 4 shows the change in heart rate displayed on the smartwatch when each pressure pattern was executed respectively at rest. From Table 4, these results showed that the displayed values were lower than the true values for all the smartwatches in the experiment at the rest environment, although these values depended on the pulse wave control pattern or subject. The results and considerations for each pattern are described in the following sections.

#### 4.3.1. The Results of Pattern A

**Figure 6:**
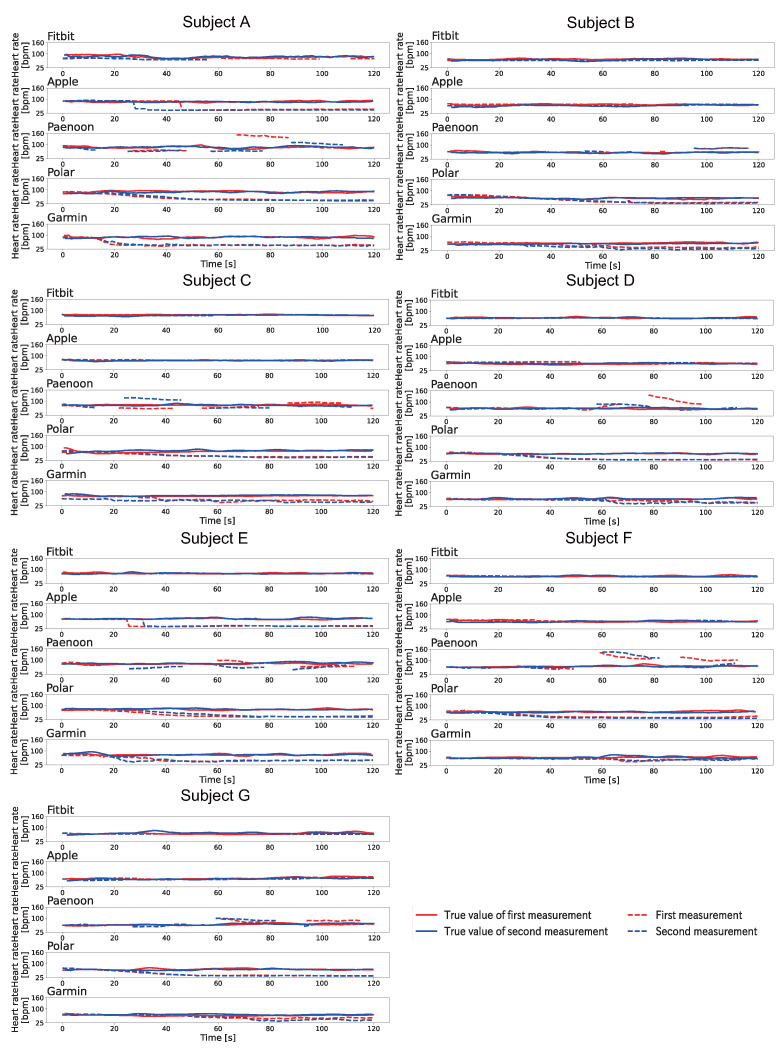
Results of the displayed heart rate when Pattern A is executed at rest. The vertical axis indicates heart rate [bpm] and the horizontal axis indicates time [s]. The red line indicates the first session, and the blue line indicates the second session. The solid line shows the correct heart rate calculated from the index finger on the left hand, and the dotted line shows the heart rate displayed on the smartwatch.

Figure 6 shows the change displayed in the heartbeat on the smartwatch when Pattern A was conducted at rest. The heart rate displayed on the smartwatch was recorded by video and manually written down frame-by-frame for 1 s at a time. The BPM was calculated based on the Equation (1). As shown in Figure 6, the heart rate displayed on the smartwatches decreased significantly for all subjects using the Polar and Garmin. Polar displayed a slowly decreased heart rate and displayed 33–46 BPM at the end of the session. Garmin also slowly decreased the displayed heart rate and displayed 36–52 BPM at the end of the session. Apple showed a significant decrease with Subject A and Subject E. Apple showed a rapid decrease in the heart rate during the session. Fitbit failed to display a significant decrease in values for all subjects. Paenoon displayed higher values than the collected values at some times.

**Figure 7:**
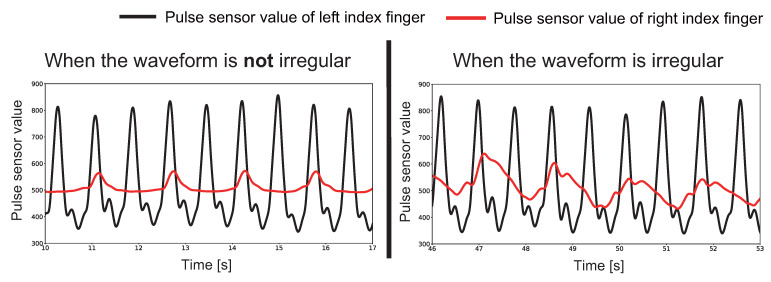
The first session of Garmin for Subject E when Pattern A was performed. The solid black line shows the pulse sensor value of the left index finger, and the solid red line shows the pulse sensor value of the right index finger. The vertical axis indicates the A/D-converted pulse wave and pressure sensor values, the horizontal axis indicates time, and the red circle indicates the pulse wave peaks. The left graph was measured at 10–17 s from the measurement start, and the right graph was measured at 46–53 s from the measurement start.

The theoretical value of Pattern A is supposed to be half of the true value. However, some sessions failed to achieve the theoretical value in some sessions. For example, the first session of the Garmin for Subject E displayed 52 BPM when the BPM was 83 BPM at the end of the session. This is because the waveform was more irregular than the natural pulse wave due to the short time between the compression and release actions. Figure 7 shows the actual waveforms measured in the first session of the Garmin for Subject E. As shown in Figure 7, the waveform of the index finger on the right hand was measured at around 15 s, since the measurement started was following Pattern A. On the other hand, the waveform of the right index finger was irregular at around 50 s from the start of the measurement. Therefore, it is necessary to improve the algorithm of the proposed method, such as the timing of compression, to make the pulse wave appear more natural. It is considered that the reason for the increase in the displayed value in the Paenoon may be the use of a threshold value in the onboard algorithm. When a peak disappears with the proposed method at the threshold setting timing, an incorrect threshold value is set. As a result, the peak could not be detected correctly. Therefore, it is necessary to perform pulse wave control based on the algorithm installed in the smartwatch.

#### 4.3.2. The Results of Pattern B

**Figure 8:**
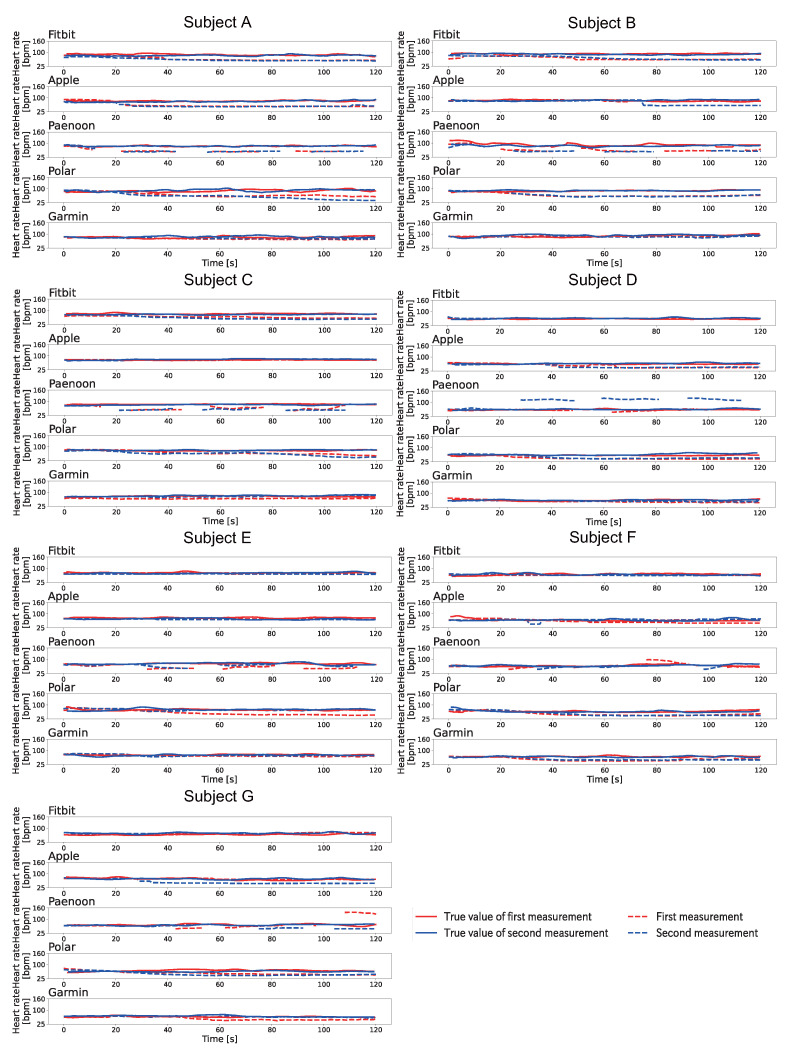
Results of the displayed heart rate when Pattern B is executed at rest. The vertical axis indicates heart rate [bpm] and the horizontal axis indicates time [s]. The red line indicates the first session, and the blue line indicates the second session. The solid line shows the correct heart rate calculated from the index finger on the left hand, and the dotted line shows the heart rate displayed on the smartwatch.

Figure 8 shows the change displayed in the heartbeat on the smartwatch when Pattern B was conducted at rest. The heart rate displayed on the smartwatch was recorded by video and manually written down frame-by-frame for 1 s at a time. The BPM was calculated based on the Equation (1). As shown in Figure 8, Fitbit showed a decrease in the heart rates for Subjects A–C. Apple showed a sudden decrease in heart rates in the second session for Subjects B, F, and G and in both sessions for Subjects A and D. Paenoon showed a sudden decrease in displayed heart rate, thereby displaying values of more than 20 BPM lower than the true values.In contrast, the second session for Subject D, the first session for Subject F, and the first session for Subject G showed that the displayed value had increased. Polar displayed 37–67 BPM values at the end of the session, except the second session for Subject E. Garmin showed a decrease of about 10–30 BPM from the true value at the end of the session in both sessions for Subjects D and E and in the first session for Subject G.

The theoretical value of Pattern B is achieved when two-thirds of the original heart rate is obtained. However, some sessions failed to achieve the theoretical values. For example, the displayed value at the end of the second session in the Polar for Subject B was 37 BPM when the true value was 94 BPM, which was very different from the theoretical value. This is because the pulse wave was disturbed by the use of the proposed method, which was the same as that used in Pattern A. Therefore, it is necessary to improve the proposed method’s algorithm so that undisturbed pulse waveforms can be measured. In addition, it is also possible that the calibration values performed before the experiment were no longer correct. Pulse waves always change depending on the user’s physical condition. The pressure value may become inappropriate due to changes in the physical condition. Therefore, it is necessary to reclibrate the pressure value when the proposed method is used continuously over a long time.

#### 4.3.3. The Results of Pattern C

**Figure 9:**
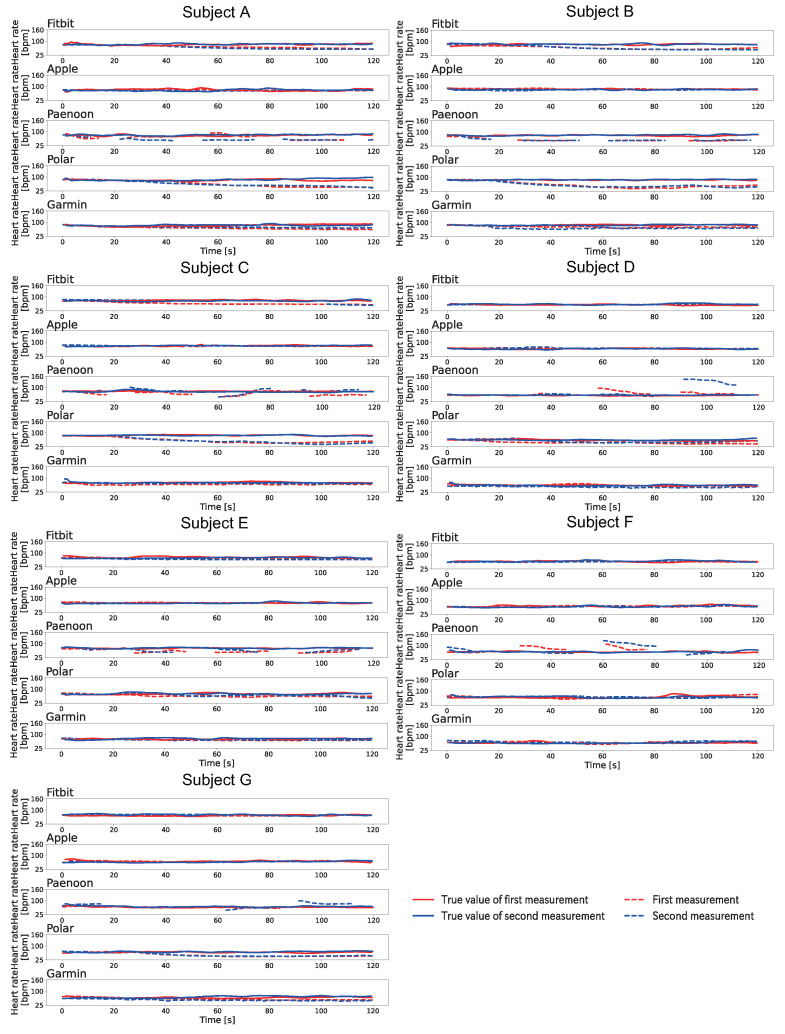
Results of the displayed heart rate when Pattern C is executed at rest. The vertical axis indicates heart rate [bpm] and the horizontal axis indicates time [s]. The red line indicates the first session, and the blue line indicates the second session. The solid line shows the correct heart rate calculated from the index finger on the left hand, and the dotted line shows the heart rate displayed on the smartwatch.

Figure 9 shows the change displayed in the heartbeat on the smartwatch when Pattern C was conducted at rest. The heart rate displayed on the smartwatch was recorded by video and manually written down frame-by-frame for 1 s at a time. The BPM was calculated based on the Equation (1). As shown in Figure 9, Polar showed a significant decrease in the displayed value, except for Subject F. The decrease trend for the displayed values was the same as in Pattern A and Pattern B. Fitbit confirmed the decrease in Subjects A–C, and it displayed a value of 55–67 BPM at the end of the session. Apple did not show a large decrease in the displayed value. Paenoon showed a decrease in the second session for Subject A, the first session for Subject C, and both sessions for Subjects B and E. In contrast, the displayed value increased from the true value at some time in the first session of Subject A, both sessions for Subject F, and the second sessions of Subjects C, D, and G. Garmin showed a decrease of 10 BPM or more at the end of the session in both sessions for Subjects A, B, and G and in the second session for Subject D.

The theoretical value of Pattern C is achieved at 1/3 of the true heart rate. However, all sessions could not display a value close to the theoretical value. This is because the theoretical heart rate is too low to be displayed, even if the pressure was correctly performed. The results in Section 4.2 show that the lower limit for each smartwatch is approximately 45 BPM. Therefore, a heart rate of 30 BPM is not expected and cannot be displayed. Therefore, it is difficult to display a heart rate close to the theoretical value of Pattern C by pulse wave control at rest; however, the displayed value can be decreased.

### 4.4. Results of Heart Rate Changes after Exercise Using Pulse Wave Control

Table 5 shows the results of sessions that changed by more than 15 BPM from the actual BPM. As shown in Table 5, these results showed that the displayed values were lower than the true values for all the smartwatches in the experiment after-exercise environment, although these values depended on the pulse wave control pattern or subject. The results and considerations for each pattern are described in the following sections.

#### 4.4.1. The Results of Pattern A

**Figure 10:**
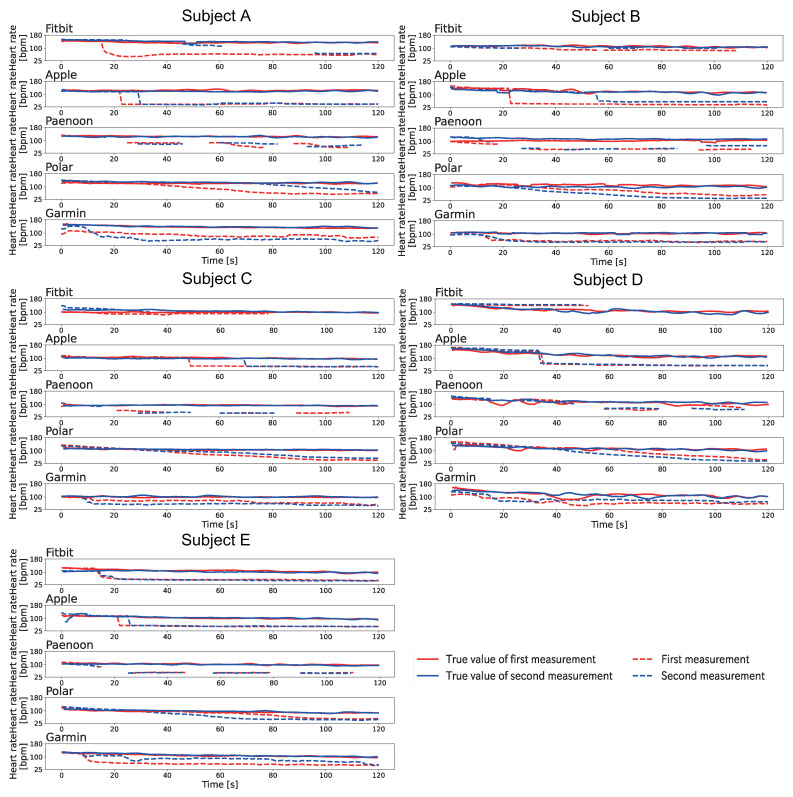
Results of the change displayed in the heartbeat on the smartwatch when Pattern A was conducted after exercise. The vertical axis indicates heart rate [bpm] and the horizontal axis indicates time [s]. The red line indicates the first session, and the blue line indicates the second session. The solid line shows the correct heart rate calculated from the index finger on the left hand, and the dotted line shows the heart rate displayed on the smartwatch.

Figure 10 shows the change displayed in the heartbeat on the smartwatch when Pattern A was conducted after exercise. The heart rate displayed on the smartwatch was recorded by video and manually written down frame-by-frame for 1 s at a time. The BPM was calculated based on the Equation (1). As shown in Figure 10, it was confirmed that the displayed value decreased in all sessions for the four models except for Fitbit. Focusing on each smartwatch, Fitbit showed a variation in results, with some sessions showing a decrease in the displayed value compared to the true value, some sessions showing an increase compared to the true value, and some sessions showing neither a decrease nor an increase. In addition, it was confirmed that the displayed value disappeared during the experiment except for the first sessions of Subject A and the both sessions of Subject E. Apple displayed 40–58 BPM at the end of the session. Apple displayed 42 BPM and 43 BPM in the sessions for Subject A, and the displayed value decreased to about one-third of the true value. Paenoon, Polar, and Garmin also showed significant decreases from the true values when the values were displayed, with some sessions recording less than half of the true value. The trend of decrease in the displayed values of all smartwatches was the same as that of the resting condition.

**Figure 11:**
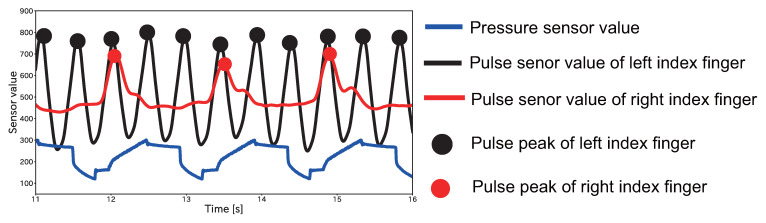
The first Apple session for Subject A when Pattern A was performed. The solid black line shows the pulse sensor value of the left index finger, the solid red line shows the pulse sensor value of the right index finger, and the solid blue line shows the pressure sensor value in the cuff. The black circle indicates the pulse wave peak of the left index finger, and the red circle indicates the pulse wave peak of the right index finger. The vertical axis indicates the A/D-converted pulse wave and pressure sensor values, the horizontal axis indicates time, and the red circle indicates the pulse peaks.

These results showed that the displayed heart rate decreased in Pattern A even after exercise. However, the value in all the sessions that showed a decrease was not half of the true heart rate, which is the theoretical value of Pattern A. Figure 11 shows the pulse sensor values and pressure sensor values for the first session of Apple of Subject A when Pattern A was performed. Figure 11 shows that there were 11 peaks in the true value of the pulse wave. However, there were only three peaks in the controlled pulse wave after passing through the device. Normally, there should be 5–6 peaks, and there were two or more disappearing peaks of the true pulse wave. This may be because the heart rate was too high, and the pressure on the upper arm did not reach its peak in time as it should have done. Figure 11 shows that the pulse wave peak in the index finger of the left hand, which was not pressured, was detected at the same time that the pressure to the cuff was ended. It is necessary that the device needs to be improved by changing to a more powerful air pump.

#### 4.4.2. The Results of Pattern B

**Figure 12:**
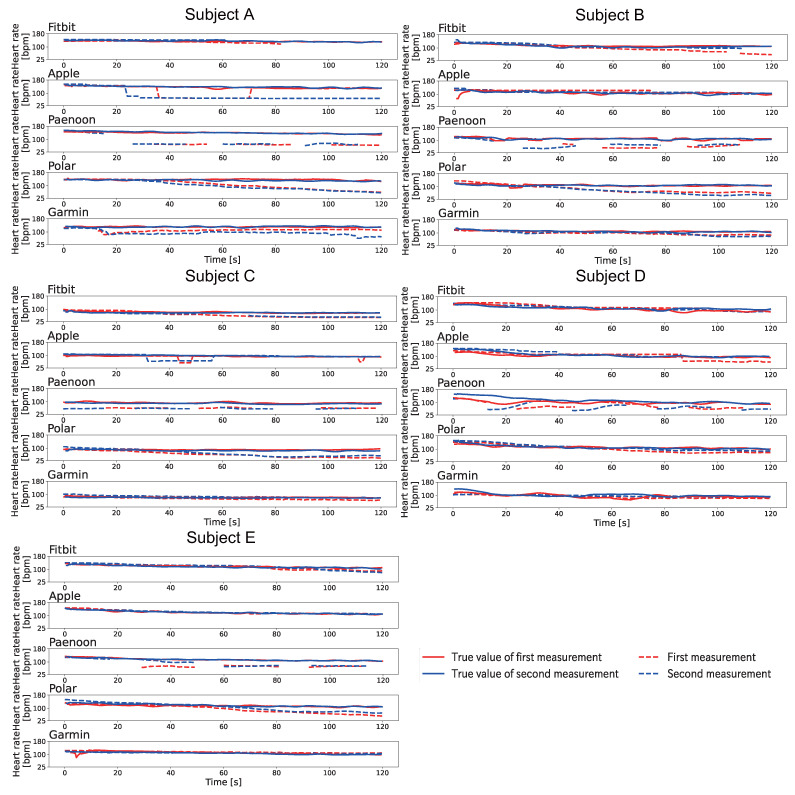
Results of the change displayed in the heartbeat on the smartwatch when Pattern B was conducted after exercise. The vertical axis indicates heart rate [bpm] and the horizontal axis indicates time [s]. The red line indicates the first session, and the blue line indicates the second session. The solid line shows the correct heart rate calculated from the index finger on the left hand, and the dotted line shows the heart rate displayed on the smartwatch.

Figure 12 shows the change displayed in the heartbeat on the smartwatch when Pattern B was conducted after exercise. The heart rate displayed on the smartwatch was recorded by video and manually written down frame-by-frame for 1 s at a time. The BPM was calculated based on the Equation (1). As shown in Figure 12, Paenoon and Polar showed a large decrease in all subjects. Polar displayed 40–88 BPM, and the displayed value varied in each session. Garmin showed a decrease of about 15–60 BPM in all sessions except the second sessions of Subjects C–E. In Apple’s second session for Subject A and the first session for Subject D, the displayed value decreased at the end of the session. In particular, 69 BPM was displayed at the end of the session in the second session of Subject A, when the true value of 131 BPM was displayed. Fitbit showed that the displayed heart rate decreased by 14–50 BPM at the end of all the sessions except for Subject A, the second session for Subject B, and the first session for Subject D. There was also a time during the session when the heart rate display disappeared.

Pattern B failed to achieve 2/3 of the theoretical true value in all sessions where a decrease was confirmed. This is because the heart rate was too high, as in Pattern A, and the upper arm was not pressed in time to reach the peak that should have been achieved. Therefore, more peaks disappeared than the set number of disappearing peaks, the theoretical value of Pattern B, and 2/3 of the true value could not be achieved.

#### 4.4.3. The Results of Pattern C

**Figure 13:**
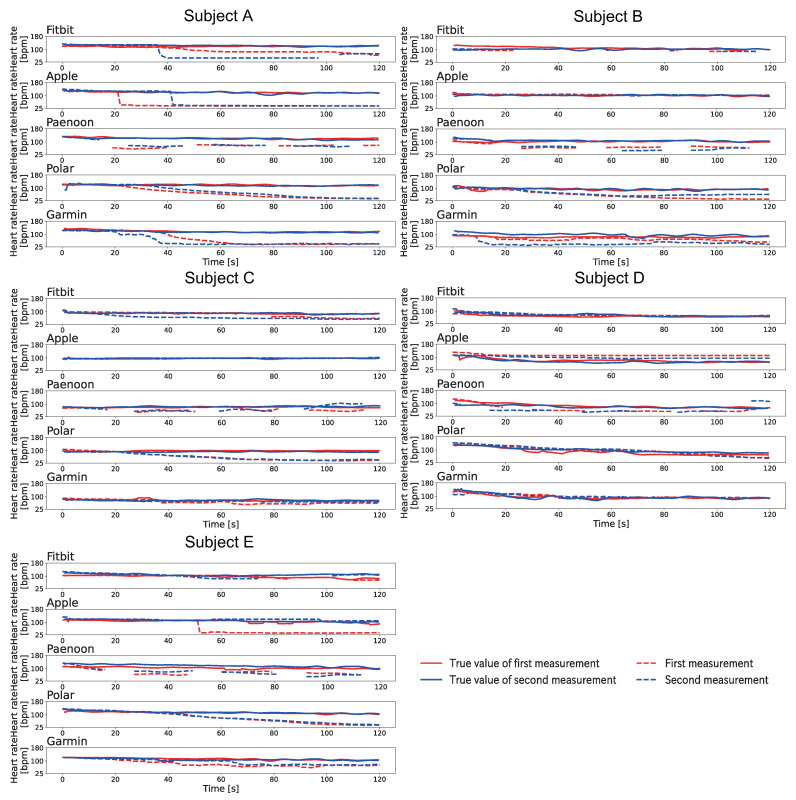
Results of the change displayed in the heartbeat on the smartwatch when Pattern C was conducted after exercise. The vertical axis indicates heart rate [bpm] and the horizontal axis indicates time [s]. The red line indicates the first session, and the blue line indicates the second session. The solid line shows the correct heart rate calculated from the index finger on the left hand, and the dotted line shows the heart rate displayed on the smartwatch.

Figure 13 shows the change displayed in the heartbeat on the smartwatch when Pattern C was conducted after exercise. The heart rate displayed on the smartwatch was recorded by video and manually written down frame-by-frame for 1 s at a time. The BPM was calculated based on the Equation (1). As shown in Figure 13, Polar showed a significant decrease in the displayed value for all subjects. Polar displayed 37–64 BPM at the end of the sessions, which varied from each session. Garmin could not show any decrease in the displayed value, which only occurred for Subject D. Fitbit showed a decrease in the displayed value for both Subjects A and C, although the heart rate display disappeared during measurement in some sessions. Apple showed a decrease only in the first session for Subject E and both sessions for Subject A. Paenoon confirmed the decrease in all sessions except in the second session for Subject C and in the second session for Subject D. In addition, the displayed value was higher than the true value in the sessions in which no decrease was observed.

Pattern C also failed to achieve 1/3 of the true value, which is the theoretical value that was confirmed to decrease in some sessions. In Pattern C, the heart rate was also too high, and the upper arm was released and pressed in time to reach the peak that it should have done. In addition, it may be that the heart rate, which cannot be displayed on the smartwatch, became a theoretical value and could not be calculated correctly by the onboard algorithm as the heartbeat calmed down over time after the end of the exercise.

## 5. Discussion

This section describes the limitations and issues of the proposed method.

### 5.1. Time for Upper Arm Pressure

The proposed method uses air pressure with a micro air pump. In this study, the time taken for pressure was approximately 0.5 s in this implemented device. From the evaluation experiment, it was confirmed that the number of peaks that disappeared more than the set number of peaks to be disappeared was lost in the after-exercise environment, as shown in Figure 11. At a resting heart rate of 60–100 BPM, which is considered the normal heart rate for humans, one peak appeared in about 0.6–1 s. The micro air pump used in this evaluation experiment was able to pressure the upper arm when at rest well in time. However, at high heart rates, such as during exercise, the heart rate increases to 120–150 BPM depending on the individual. This means that one peak appeared at 0.4–0.5 s, and the micro air pump used in this evaluation experiment could not pressurize the upper arm well in time. Therefore, it is better to use a high-performance micro air pump that can pressurize in time, even at high heart rates. Nevertheless, the higher the performance, the larger the size of the pump is expected to become, which may make it difficult to carry around.

### 5.2. The Pulse Waveform When the Proposed Method Was Used

From the results of the evaluation experiment, a waveform that was not similar to a natural pulse waveform was output when pressure was applied and reduced over a short time. This behavior was not seen immediately after the start of measurement but started to appear about 40 s after the start of measurement. In an environment such as this evaluation experiment, where only the displayed heart rate value on the smartwatch was seen, there was no way to know that the proposed method was used since the waveforms were not seen. However, in an environment where the pulse waveform is checked itself, the use of the proposed method can be judged quickly. It is necessary to make a natural pulse waveform measurement even when using the proposed method in order to improve the proposed method.

### 5.3. Pulse Wave Measurement Position

In this evaluation experiment, a pulse wave sensor was attached to the index finger of the left hand, which was not influenced by pressure. Originally, it was thought to be better to attach the pulse wave sensor to the same upper arm as the side that presses the upper arm, which reduces the burden on the user because the pulse wave sensor can be attached at the same time as the cuff is attached. However, it is not possible to obtain a pulse waveform even if a pulse sensor is attached to any part of the upper arm.

In addition, the pulse sensor value contains noise when the contact condition between the LED or photodiode and the user’s skin changes. The sensor values cannot be measured stably when the motion is strong, such as during exercise. Therefore, it is necessary to determine the sensor-wearing position that is stable even during exercise. If the pulse wave is acquired at a different position, there is a time difference from the pulse wave measured at the upper arm due to the PWV (pulse wave velocity). It should also be evaluated whether it is possible to adjust for the time difference and calculate the appropriate starting time of the pressure.

### 5.4. Experiment Subjects

In the experiments of this paper, only young males participated. As a result, the proposed method was confirmed to be effective when young men were the users. However, it is not only young men who may use the proposed method. For example, it is assumed that various users, such as women, older people, and low blood pressure users, may use the proposed method. We think that the proposed method can be used regardless of gender, as the proposed method uses data obtained from a pulse wave sensor for upper arm pressure. On the other hand, when users with a low heart rate, such as elderly people of older age, use the proposed method, it is assumed that the pulse wave data cannot be measured for controlling the cuff. When pulse wave data cannot be measured, the proposed method may not work correctly, and the pulse wave control may not be correct. Therefore, it is necessary to investigate the usefulness of the proposed method by conducting experiments on a wide range of subjects, taking into account their age, blood pressure, and body shape.

### 5.5. Impact on the Real-World Scenario

Currently, many systems using biometric information are available. The system provider cannot know the state of the measured sensor values, and even if a malicious user uses the proposed method, the system provider cannot judge that the system was used. Here, we take the example of a health insurance service business. Some health insurance systems require the submission of heart rate data measured by smartwatches specified by providers such as Apple and Fitbit. Based on the submitted heart rates, the insurance company determines the health status of the user, and the insurance company offers a discount on insurance premiums if the user is healthy. In other words, a malicious user could use the proposed method to create a fake healthy heart rate and submit the data, and the system provider could still provide a discount on insurance premiums in case no countermeasures were taken by the system provider. Compared to the user who pays the same amount of insurance, the user who submits a fake heart rate is judged to be healthy and may receive more insurance benefits when the user becomes sick. This means that the system provider will pay more in insurance payments to users and will maintain its business model by increasing premiums and cutting the rate of return on discounts and services. As a result, all system users are disadvantaged by premium increases, among other factors, and the system provider can maintain its business by reducing the damage through price increases, among other factors. The use of the proposed method by the malicious user is thought to lead to the disadvantage of all users. Therefore, the impact of the proposed method on real-world scenarios when it becomes popular in a general way is a problem that cannot be ignored.

In the future, it is necessary to develop a method to detect the use of the proposed method as a countermeasure against the use of the proposed method. For example, the shape of the pulse waveform may differ between the normal pulse waveform and the pulse waveform during an attack. Based on this, it is possible that attacks can be detected using waveform matching or deep learning. Also, it is considered that blood pressure information can be used. There is a report that blood pressure increases as the heart rate increases [32]. In other words, it is possible to detect an attack when the heart rate is low despite high blood pressure.

## 6. Conclusions

In this study, we proposed an upper arm pressing device that misleads the pulse wave sensor in a smartwatch to clarify the possibility of attacks on wearable devices. In addition, we evaluated whether the implemented device could misrecognize the heart rate application installed in the smartwatch using the proposed method. Eight subjects wore five different models of smartwatches, and three pressure patterns were performed in two different environments: at rest and after exercise. For Pattern A at rest, Polar and Garmin showed a decrease in the value displayed on the smartwatch from the true value for all subjects. Polar showed 33–46 BPM at the end of the measurement, and Garmin showed 36–52 BPM at the end of the measurement. For Pattern B at rest, Polar displayed a decrease in values on the smartwatches compared to the true values except for the second session for Subject E. At the end of the measurement, 37–67 BPM was displayed. For Pattern C at rest, Polar showed a decrease in the value displayed on the smartwatch compared to the true value for all subjects, with the maximum difference from the true value being 55 BPM. For Pattern A after exercise, the value displayed on the smartwatch was decreased from the true value for all subjects in all four models except Fitbit. All smartwatches showed a greater decrease than the true value. For Pattern B after exercise, Paenoon and Polar showed a decrease in the value displayed on the smartwatches compared to the true value for all subjects. In particular, Polar displayed 40–88 BPM at the end of the measurement, which varied from subject to subject. For Pattern C after exercise, Polar showed a decrease in the value displayed on the smartwatch from the true value for all subjects. Polar displayed a value of 37–64 BPM at the end of the measurement. From these results, it was confirmed that the displayed heart rate on the smartwatch decreased from the true value in all models, depending on the pressure control pattern, both in the resting and after-exercise environments.

In the future, the small size of the device will be considered so that it can be carried around. In addition, instead of misusing pulse wave manipulation as was done in this study, we plan to propose interaction techniques and other technologies that make use of pulse wave manipulation.

## Figures and Tables

**Table 1:** Execution procedure and content in calibration phase.

Order	Name of the Procedure	Description
1	Data measurement	· Pulse wave and pressure are measured every *T* [ms]. (*T* is a value that can be set arbitrarily.)
2	Pressure start	· The solenoid valve is closed. · The micro air pump starts to pump air to the cuff. · The pressure is applied to the cuff until $x_{air\_max}$ is satisfied. (at the start of the calibration phase, $x_{air\_max}=400$.)
3	Keep pressure	· The air supply from the micro air pump is stopped. (when $x_{air}\left(t\right)\geq x_{air\_max}$ is satisfied.) · The pressure value in the cuff is kept.
4	Pulse wave peaks detection	· The peaks in the measured pulse wave are detected. · The peak detection algorithm [21] is used. · The pulse wave disappearing state is determined when the peak is not detected for 2 s.
5	Pressure end	· The solenoid valve is opened for 1 s. · The air pressure value in the cuff is reduced.
6	Standby for next pressure	· The value of $x_{air\_max}$ is updated. · When the pulse wave disappearing state is detected, $x_{air\_max}=x_{air\_max}-20$, and return to Data measurement. · When the pulse wave disappearing state is not detected, $x_{air\_max}=x_{air\_max}+20$, and the calibration phase is finished.

**Table 2:** Execution procedure and content in pulse wave control phase.

Order	Name of the Procedure	Description
1	Data measurement	· Pulse wave and pressure are measured every *T* [ms]. (*T* is a value that can be set arbitrarily.)
2	Pressure start	· The solenoid valve is closed. · The micro air pump starts to pump air to the cuff. · The pressure is applied to the cuff until $x_{air\_max}$ is satisfied.
3	Pressure keeping	· The air supply from the micro air pump is stopped. (when $x_{air}\left(t\right)\geq x_{air\_max}$ is satisfied.) · The pressure in the cuff is kept until $N_{stop}$ peaks are detected. (the number of disappearing pulse wave peaks is set to $N_{stop}$.)
4	Pulse wave peaks detection	· The peaks in the measured pulse wave are detected. · The peak detection algorithm [21] is used.
5	Pressure end	· After detecting $N_{stop}$ peaks, the solenoid valve is opened. · The pressure value is reduced until reaching $x_{air}\left(t\right)=x_{air\_max}\times 0.5$.
6	Standby for next pressure	· The pressure value is kept until detecting $N_{appear}$ peaks. (the number of appearing pulse wave peaks is set to $N_{appear}$.) · Return to Data measurement if the process wants to repeat.

**Table 3:** A list of the highest and lowest heartbeats displayed on the smartwatch. “Nan” indicates that the value was not displayed.

Smartwatch	Maximum		Minimum	
	Input	Output	Input	Output
Fitbit	210	201	55	54
Apple	220	210	45	44
Polar	Nan	Nan	Nan	Nan
Garmin	240	189	30	45
Paenoon	190	180	48	48

**Table 4:** The result of the changes in displayed heart rate on the smartwatches at rest environment. The ↘ indicates that the value displayed on the smartwatch decreased by more than 15 BPM below the true value, and ↗ indicates that the value displayed on the smartwatch increased by more than 15 BPM above the true value. No mark indicates that the smartwatch’s value was unchanged.

Pattern	Subject	Session	Smartwatch				
			Fitbit	Apple	Paenoon	Polar	Garmin
A	A	1st		↘	↗	↘	↘
		2nd		↘	↗	↘	↘
	B	1st			↗	↘	↘
		2nd			↗	↘	↘
	C	1st			↗	↘	↘
		2nd			↗	↘	↘
	D	1st			↗	↘	↘
		2nd			↗	↘	↘
	E	1st		↘	↗	↘	↘
		2nd		↘	↘	↘	↘
	F	1st			↗	↘	↘
		2nd			↗	↘	↘
	G	1st			↗	↘	↘
		2nd			↗	↘	↘
B	A	1st	↘	↘	↘	↘	
		2nd	↘	↘	↘	↘	
	B	1st	↘		↘	↘	
		2nd	↘	↘	↘	↘	
	C	1st	↘		↘	↘	
		2nd	↘		↘	↘	
	D	1st		↘	↘	↘	↘
		2nd		↘	↗	↘	↘
	E	1st			↘	↘	
		2nd			↘		
	F	1st		↘	↗	↘	↘
		2nd			↘	↘	↘
	G	1st			↗	↘	↘
		2nd		↘	↘	↘	
C	A	1st	↘		↗	↘	↘
		2nd	↘		↘	↘	↘
	B	1st	↘		↘	↘	↘
		2nd	↘		↘	↘	↘
	C	1st	↘		↘	↘	
		2nd	↘		↗	↘	
	D	1st			↗	↘	
		2nd			↗	↘	↘
	E	1st			↘	↘	
		2nd			↘	↘	
	F	1st			↗		
		2nd			↗		
	G	1st				↘	↘
		2nd			↗	↘	↘

**Table 5:** The result of the changes in displayed heart rate on the smartwatches after exercise environment. The ↘ indicates that the value displayed on the smartwatch decreased by more than 15 BPM below the true value, and ↗ indicates that the value displayed on the smartwatch increased by more than 15 BPM above the true value. No mark indicates that the smartwatch’s value was unchanged.

Pattern	Subject	Session	Smartwatch				
			Fitbit	Apple	Paenoon	Polar	Garmin
A	A	1st	↘	↘	↘	↘	↘
		2nd	↘	↘	↘	↘	↘
	B	1st	↘	↘	↘	↘	↘
		2nd		↘	↘	↘	↘
	C	1st		↘	↘	↘	↘
		2nd		↘	↘	↘	↘
	D	1st	↗	↘	↘	↘	↘
		2nd	↗	↘	↘	↘	↘
	E	1st	↘	↘	↘	↘	↘
		2nd	↘	↘	↘	↘	↘
B	A	1st			↘	↘	↘
		2nd		↘	↘	↘	↘
	B	1st	↘		↘	↘	↘
		2nd			↘	↘	↘
	C	1st	↘		↘	↘	↘
		2nd	↘		↘	↘	
	D	1st		↘	↘	↘	
		2nd	↘		↘	↘	
	E	1st	↘		↘	↘	
		2nd	↘		↘	↘	
C	A	1st	↘	↘	↘	↘	↘
		2nd	↘	↘	↘	↘	↘
	B	1st			↘	↘	↘
		2nd			↘	↘	↘
	C	1st	↘		↘	↘	↘
		2nd	↘		↗	↘	↘
	D	1st		↗	↘	↘	
		2nd		↗	↗	↘	
	E	1st		↘	↘	↘	↘
		2nd		↗	↘	↘	↘

## Data Availability

The data are not publicly available due to using biometric information.
